# Antigen targeting to M cells for enhancing the efficacy of mucosal vaccines

**DOI:** 10.1038/emm.2013.165

**Published:** 2014-03-14

**Authors:** Sae-Hae Kim, Yong-Suk Jang

**Affiliations:** 1 Department of Molecular Biology, Institute for Molecular Biology and Genetics, Chonbuk National University, Jeonju, Korea; 2 Department of Bioactive Material Sciences, Research Center of Bioactive Materials, Chonbuk National University, Jeonju, Korea

**Keywords:** immunity, M cell, vaccine

## Abstract

Vaccination is one of the most successful applications of immunology and for a long time has depended on parenteral administration protocols. However, recent studies have pointed to the promise of mucosal vaccination because of its ease, economy and efficiency in inducing an immune response not only systemically, but also in the mucosal compartment where many pathogenic infections are initiated. However, successful mucosal vaccination requires the help of an adjuvant for the efficient delivery of vaccine material into the mucosa and the breaking of the tolerogenic environment, especially in oral mucosal immunization. Given that M cells are the main gateway to take up luminal antigens and initiate antigen-specific immune responses, understanding the role and characteristics of M cells is crucial for the development of successful mucosal vaccines. Especially, particular interest has been focused on the regulation of the tolerogenic mucosal microenvironment and the introduction of the luminal antigen into the lymphoid organ by exploiting the molecules of M cells. Here, we review the characteristics of M cells and the immune regulatory factors in mucosa that can be exploited for mucosal vaccine delivery and mucosal immune regulation.

## Introduction

The Sabin live attenuated oral polio vaccine introduced in 1950 is an example of a successful mucosal vaccination.^1^ In addition to its convenience, using mucosal routes, especially the oral route, can boost the economic value of vaccines and make needle-free delivery possible, allowing for safe and easy vaccine administration by personnel without medical training.^2^ More importantly, mucosal vaccination can induce the immune response systemically as well as in mucosal surfaces, which is poorly induced in parenteral immunization.^3^ Mucosal epithelium covering the aerodigestive and urogenital tracts constitute a special immune system that maintains mucosal homeostasis by restricting the influx of luminal antigens and dominantly inducing tolerance.^4^ Considering that 90% of infections occur in mucosal areas, it is conceivable that using mucosal vaccinations to establish protective immunity in this frontline of pathogen infection could overcome some of the limitations of current injection-based vaccines.^5^ Nevertheless, only a few commercial mucosal vaccines are currently available, including FluMist, NASOVAC, RotaTeq, Vivotif, Orochol and Dukoral.^6^ The limited availability of mucosal vaccines may be closely related with the poor understanding, until recently, of the mucosal immune system and the lack of effective and clinically acceptable mucosal vaccine adjuvants. However, recent research progress on the mucosal immune system and mucosal vaccine adjuvants makes it possible for us to consider mucosal vaccines as plausible alternatives to parenteral vaccination.^7^ In this review, we summarize the characteristics of M cells, which are involved in antigen uptake and other critical elements of the mucosal immune system, and strategies to improve the efficiency of mucosal immune response induction.

## Oral mucosal vaccine

For almost a century, many studies have concentrated on developing oral vaccines against enteric pathogens as the route of immunization is critical in the successful induction of the immune response in the mucosal compartments where the infections initiate.^8^ Nevertheless, only a handful of licensed oral vaccines against these enteric infections exist, as summarized in Table 1.^8^ To prevent the infection of *Vibrio cholera*, one of the causative organisms of diarrhea, establishing the mucosal immune responses requires the induction of anti-bacterial or anti-cholera toxin (CT) SIgA in the gut.^9^ Dukoral, which consists of recombinant cholera toxin B subunit (CTB) and inactivated *V. cholera*, is an internationally licensed oral cholera vaccine.^6^ This vaccine shows not only a high protective capability against *V. cholera* infection in the first year after oral vaccination, but also cross-protection against heat-labile enterotoxin-producing enterotoxigenic *Escherichia coli* because of the CTB component included in the vaccine.^9, 10^ A second licensed oral vaccine against CT (ORC-Vax or Shanchol) consists of inactivated *V. cholera* and formalin-killed O139 bacteria without the CTB component.^11^ The protective effect of oral cholera vaccine was verified in a recent outbreak of cholera in Hanoi, Vietnam.^12^ *Salmonella typhi* causes 200 000 deaths per year worldwide and is a good target for mucosal vaccine development because the bacterial infection initiates in the mucosa of the small and large intestines and spreads to the blood.^13^ To protect against this pathogen, the induction of both mucosal IgA to block the interaction of the bacteria with the intestinal epithelium and serum IgG to inhibit the spreading of the pathogen into the systemic compartment is required.^14^ Vivotif, an oral Typhoid vaccine, was developed in the 1970s and is currently commercialized in enteric-coated capsules containing lyophilized Ty21a (Ty21a/Vivotif).^14^ Although this vaccine only shows about 60% protective efficiency, which might be due to the induction of oral tolerance and problems in vaccine formulation, it induces mucosal IgA, systemic IgG and cytotoxic T-lymphocyte responses.^15, 16^ Finally, rotavirus, a causative agent of diarrhea, is responsible for the death of 453 000 children each year, with >80% of the deaths occurring in developing countries.^17^ Two types of oral live attenuated rotavirus vaccines, Rotarix and RotaTeq, induce high protective immunity against viral infection through the induction of mucosal IgA and systemically neutralizing IgG.^18, 19^

## Basic principles of mucosal vaccination

**Figure 1:**
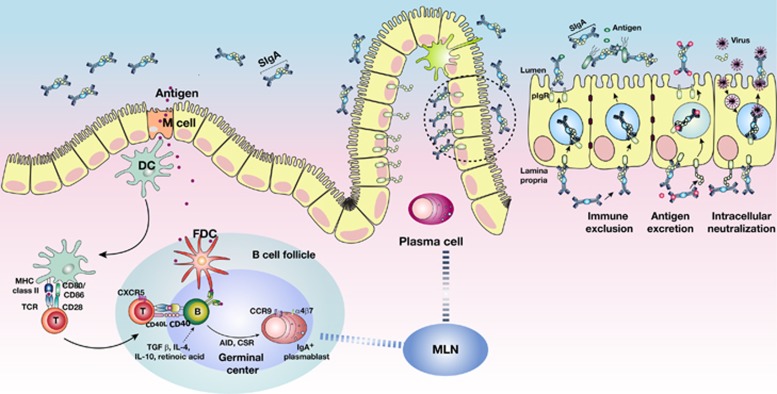
Schematic diagram of mucosal immune induction to generate T-cell-dependent IgA production. In PP, antigens transcytosed by M cells encounter DCs in the subepithelial dome. DCs loaded with the antigens migrate into the interfollicular T-cell zones and induce the conversion of naïve T cells into effector T cells. Antigen-specific effector CD4^+^ T cells that express CD40 ligand enable IgA class-switch recombination through the interaction with B cells expressing CD40 receptors in their B-cell follicle and the secretion of cytokines such as IL-4 and IL-10, which induces the expression of activation-induced cytidine deaminase. IgA^+^ plasmablasts, home to the mucosal effector site and the dimeric IgA produced from IgA^+^ plasma cells, are transcytosed to the intestinal lumen as SIgA by interacting with the polymeric Ig receptor.

Mucosal vaccination initiates by introducing exogenous antigens into the mucosa-associated lymphoid tissue that is defined as solitary-organized mucosa-associated lymphoid follicles and characterized as lacking afferent lymphatics.^20^ Therefore, antigen-specific mucosal immune induction depends on the initial taking up exogenous antigens.^21^ In subepithelial dome, dendritic cells (DCs) loaded with introduced antigens migrate to intrafollicular T-cell areas and activate the T cells, which, in turn, facilitate IgA class switch recombination and somatic hypermutation in B cells through the CD40-CD40 ligand signaling pathway.^22^ Eventually, in the mucosal effector region, the antigen-specific dimeric IgA produced from IgA-expressing plasma cells is transported to the lumen via polymeric Ig receptors and becomes SIgA, which has a role in the first line of protection through immune exclusion, intracellular neutralization and antigen excretion (Figure 1).^23^ One important point to be considered in mucosal vaccination is that the effector sites for antigen-specific SIgA immune responses are clearly correlated with the routes of mucosal immunization.^24^ Intranasal immunization induces SIgA in the upper respiratory, gastric and genital tracts by initiating the immune response in nasopharynx-associated lymphoid tissue, whereas oral immunization induces the IgA response in the gastrointestinal tract and the salivary and mammary glands by introducing the antigen into the Peyer's patches (PPs) of the gut-associated lymphoid tissue.^4^ For example, when the CTB subunit (WC/rBS) is delivered via the nasal route into humans, CTB-specific IgA is strongly induced in the nasal cavity, large intestine and genital tracts but not in the small intestine and salivary glands.^25, 26^ On the other hand, the immunization of the same antigen via the oral route highly evokes antigen-specific IgA production in the small intestine and salivary glands but not in the nasal cavity.^27^ Consequently, it is important to select the proper application route for mucosal vaccinations according to the infection route. For example, mucosal vaccines against influenza virus, such as FluMist, are applied through the intranasal route, but mucosal vaccines for rotavirus, poliovirus, *S. typhi* and *V. cholera* are delivered via the oral route.^28^ The characteristics of the relationship between the routes of immunization and the effective compartments for mucosal immunization are summarized in Table 2.

Despite the successful development of a few oral mucosal vaccines, the number of currently available oral vaccines is very limited compared with the number of parenteral vaccines, as summarized in Table 1. This limited availability of oral mucosal vaccines is closely related with the lack of an effective antigen delivery system and a strong adjuvant to stimulate immunity because of the intrinsic nature of the mucosal immune system, which has a low efficiency in antigen delivery into the inductive site and a tendency to induce oral tolerance.^36^ Consequently, research interests in developing effective mucosal vaccines have been concentrated on M cells because characteristics such as high transcytotic activity and intracellular pockets containing various antigen-presenting cells make M cells important effectors in antigen delivery and initiators of antigen-specific mucosal immunity.

## Unique features of M cells for antigen influx and initiation of immune response

**Figure 2:**
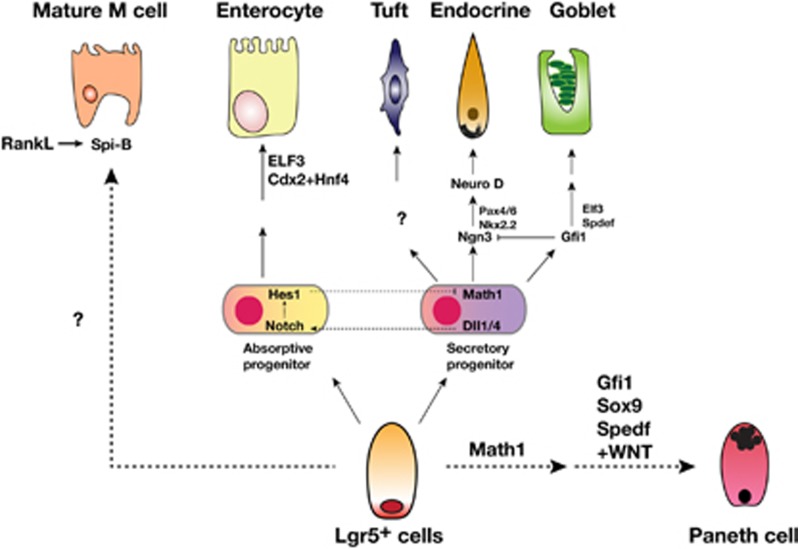
Events in differentiation of the crypt base columnar cells to intestinal epithelial cells (modified from reference 38).

The small intestinal epithelium is consisted of six differentiated epithelial cell types including goblet cells, Paneth cells, enteroendocrine cells, tuft cells, enterocytes and M cells.^37^ The follicle-associated epithelium (FAE) of PP is mainly composed of absorptive enterocytes and M cells compared with villous epithelium, which contains several secretory lineage cells such as Paneth cells, goblet cells and enteroendocrine cells (Figure 2).^38^ The composition of FAE is closely associated with sampling of luminal antigens and induction of antigen-specific immune response.^39^ Especially, M cells represent the unique feature of FAE in PP and are responsible for initiation of antigen-specific immune response.^40^ M cells were first identified from rabbit appendix in 1965 and initially called lymphoepithelial cells.^41^ Later, it was renamed microfold (M) cells owing to the observed ‘microfold' structure on apical surface of the cells from human.^41^ Morphological features of M cells make it possible to distinguish M cells from enterocytes such that apical surface of M cells are shown as down in the hollow because of short and irregular microvilli.^40^ Along with this feature, thin glycocalyx layer of M cells make them attractive target for luminal antigen influx and bacterial attachment.^42^ The basolateral pocket structure shown in M cells not only represents the morphological characteristics of the cell but also is closely associated with mucosal immune induction through interaction with several immune cells including B cells, T cells, macrophages and DCs, which are localized within the pocket.^42^

## The origin and development of M cells

The origin of M cells in FAE of PP was unclear until recently.^43^ It was identified by ‘lineage tracing' that Lgr5 (leucine-rich repeat-containing G protein-coupled receptor 5)-expressing stem cells in crypt are the origin of all intestinal epithelial cells and, in addition, a single Lgr5^+^ stem cell can be organized to the mini-gut.^44^ Lgr5 encodes a serpentine receptor and is a marker for crypt base columnar stem cells, which are continuously cycling. In addition, Lgr5 is closely associated with Wnt signaling.^45^ It is known that transcription factors in Lgr5^+^ stem cells have critical roles in deciding cell fates (Figure 2).^38^ For instance, repressing the expression of Math1 and Hes1 by Notch signaling pathway decides the fate of the enterocytes.^38^ In addition, goblet cell formation is dependent on transcriptional factor sterile α-motif (SAM)-pointed domain-containing Ets-like factor (SPDEF).^46^ The transcription factor responsible for M cells differentiation was characterized by the comparison of mRNA profiles between FAE and M cells after the treatment of receptor activator of nuclear factor kappa-B (RANK) ligand (RANKL). It was suggested that the Ets family transcription factor Spi-B regulates M-cell maturation in RANKL-dependent manner.^47^ When mini-gut originated from normal Lgr5^+^ stem cells was treated with RANKL, mature M cells expressing M cell-specific molecule glycoprotein-2 (GP2) were developed, but not in the mini-gut formed from Spi-B-deficient stem cells.^48^ RANKL treatment of villous epithelium and small intestine organoid enhanced the expression of *Gp2*, *Spi-b*, *Annexin A5*, *C motif chemokine ligand 20*, *tumor necrosis factor*, *alpha-induced protein 2* (*M-Sec*), *C-C motif chemokine ligand 9* (*Ccl9*), *Prion protein* and *MARCKS-like 1*.^49^ Among them, the expression of GP2 and CCL9, markers of mature M cells, is regulated by Spi-B, and the effect of CCL20 and CCR6 signaling on M-cell maturation was already confirmed.^49^ Also, it is suggested that M-cell patterning among the FAE is closely associated with Notch and the ligand, jagged-1, signaling.^50^ Although M cells can be differentiated from only Lgr5^+^ stem cells by RANKL treatment, other factors are also suggested to be involved in M-cell differentiation because M cells, which were identified with M cell-specific antibody NKM 16-2-4, were described even in Spi-B knock-out mouse.^51^ For example, signaling through CD137 or macrophage migration inhibitory factor produced by interaction between B cells and M-cell progenitors may also induce the functional M cells maturation. In addition, pathogenic bacteria promote M-cell differentiation by inducing CCL20 expression or EMT-regulating transcription factor Slug.^49, 52^

## Enhancing the efficiency of oral mucosal vaccines via M cell-targeting of the antigen

**Figure 3:**
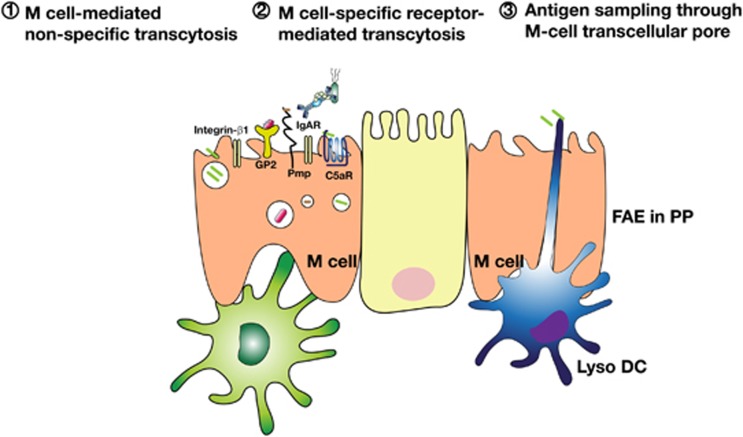
Three possible pathways proposed for luminal antigen sampling by PP M cells. First, M-cell-mediated nonspecific transcytosis occurs through clathrin-coated vesicle formation, actin-dependent phagocytosis or macropinocytosis. Second, specific receptor-mediated transcytosis has a role as immune surveillance sensor because it can be exploited by pathogens such as *Salmonella*, *Yersinia* and *Brucella*. Third, Lyso DCs, which have strong phagocytic activity and antigen sampling ability, localized in M cells extend their dendrite into lumen to take up antigens.

For a long time, developing oral vaccines that target M cells has been difficult because of the limited understanding of surface molecules in the apical area of M cells. Consequently, there was not enough information on the specific markers of M cells.^53^ This difficulty was partly overcome by establishing an *in vitro* human M-like cell culture model and identifying an M cell-specific antibody, NKM 16-2-4, generated by the immunization of UEA-1^+^ WGA^−^ cells.^54, 55^ Several M cell-specific molecules that have been identified are summarized in Table 3. For example, the M cell-targeting ligand Co1 was selected by biopanning a phage display library against human M-like cells, suggesting the expression of a complement 5a receptor (C5aR) on M cells that was confirmed in mouse M cells.^59, 72^ Transcriptomic profiling studies also suggest that genes for glycoprotein-2 (*Gp2*), tumor necrosis factor-α expressed-induced protein 2 (*Tnfaip2*) and *Ccl9* are expressed in mature M cells.^73, 74^ In addition, recent research progress on M cells suggested three pathways for luminal antigen sampling by M cells; nonspecific endocytosis, specific receptor-mediated endocytosis and via extension of transcellular dendritic processes by Lyso DCs, which have strong phagocytic activity and antigen sampling ability (Figure 3).^39^ Interestingly, the growing knowledge on M cell-specific markers supports the idea that antigen uptake in M cells occur via specific receptors that are closely related with pathogenic infection in these cells.^36^ In fact, it is easily conceivable that marker molecules expressed on M cells can be exploited by pathogens as their entry sites. With M-cell deficiencies, oral infections by *Yersinia enterocolitica*, prions and retroviruses do not occur. *Listeria monocytogenes*, *S. typhimurium*, poliovirus and reovirus also prefer M cells as the portal for their infection.^70, 75^ Interestingly, some pathogens enter their host by interacting with molecules expressed on M cells (Table 3).^60^ For instance, type I reovirus specifically targets M cells through the interaction between σ1 protein and α (2, 3) sialic acid residues, although this glycosylation pattern is abundantly detected in all host epithelium.^62^ In the case of FimH^+^, a component of type I pili expressed on Gram-negative bacteria such as *E. coli* and *S. typhimurium* infection into M cells is dependent on its interaction with the GP2 protein expressed on the apical area of M cells.^61^ Similarly, the infection of *Y. enterocolitica* is closely related with C5aR on M cells, whereas the infection of *Brucella abortus* depends on a cellular prion protein on M cells.^59, 67^ Based on these observations, it is plausible to use M cell-specific surface markers and receptors for the effective delivery of vaccine materials into the host. For example, when the M cell-specific antibody NKM 16-2-4, which recognizes α (1, 2) fucose-containing carbohydrates, was applied to an oral vaccine model against botulinum toxin, the NKM 16-2-4 combined antigen targeted M cells with high efficiency and induced antigen-specific IgA.^54^ In our own studies with an oral dengue virus vaccine model using M cell-targeting Co1 ligand or C5aR ligand OmpH, oral immunization with the Co1- or OmpH-conjugated EDIII protein of DENV-2 not only enhanced the M cell targeting of EDIII protein through its interaction with C5aR on M cells, but also evoked the induction of both antigen-specific neutralizing IgG in the serum and SIgA in fecal extract.^59, 72^

## Stimulation of innate and adaptive immunity by mucosal adjuvants

Oral tolerance implies immune unresponsiveness against orally introduced antigens in both mucosal and systemic compartments.^76^ When we consider the oral mucosal environment, which is continuously exposed to enormous amounts of antigens such as components of food and microorganisms, oral tolerance exerts a pivotal role in immune homeostasis of the mucosa.^77^ In the case of vaccination, this system should be overcome using adjuvants to enhance the immunity against the introduced vaccine materials by modulating the innate and adaptive immunity. The tolerogenic mucosal environment can be induced to adopt an inflammatory environment through the activation of the innate immune response by bacterial components.^21^ However, a safe and effective licensed mucosal vaccine adjuvant is not currently available (Table 4).^8, 75^ TLR agonists such as muramyl dipeptide, monophosphoryl lipid A and flagellin are main candidates for mucosal vaccine adjuvants because they are able to link innate and adaptive immune reactions.^21, 79^ However, their application in oral vaccines may evoke unwanted adverse effects such as the induction of inflammation and autoimmunity.^83^ Bacterial toxins have also been considered for mucosal adjuvants. For example, CTA1-DD has been suggested to be a safe and effective adjuvant. CTA1-DD can activate the complement system and consists of D-fragments from *S. aureus* protein and the A and A1 portions of CT. In follicular DCs, the interactions between CTA1-DD and complement receptor CD21 enhance the formation of the germinal center, resulting in the development of high-affinity IgA and memory B cells. At the same time, these interactions evoke Th1, Th2, Th17 and cytotoxic T-lymphocyte immunity through DC activation.^80^ *Quillaja* saponins and cationic DDA have shown promising activity as mucosal adjuvants, although their mechanisms of action are poorly understood.^84^ In addition, ligands for M cell-specific markers that are exploited by pathogens could have roles as mucosal adjuvants through their enhancement of T-cell immunity without the induction of oral tolerance.^53^ For example, the outer membrane protein H of *Y. enterocolitica* interacts with C5aR on M cells and can not only enhance antigen delivery to the mucosal immune inductive site, but can also enhance the induction of antigen-specific immune responses in systemic and mucosal compartments.^59^

## Conclusions and future perspectives

The necessity to develop oral mucosal vaccines has been widely recognized. In order to develop successful oral mucosal vaccines, it is essential to understand the mechanisms of luminal antigen sampling in M cells and to identify effective mucosal adjuvants. We are now in good position to utilize mucosal immune compartment for delivering vaccine materials to take advantage of oral mucosal vaccines. We expect that the study of M cell-targeting receptors exploited by pathogens can provide valuable information that can advance both antigen-targeting and mucosal adjuvants.

## Figures and Tables

**Table 1:** List of currently licensed mucosal vaccines (modified from reference 8)

*Pathogens*	*Trade names*	*Composition*	*Dosage*	*Immunological mechanism*	*Efficacy*
Rotavirus	Rotarix; RotaTeq	Live attenuated, monovalent or pentavalent rotaviruses	Oral, 3 doses	Mucosal IgA and systemic neutralizing IgG	Over 70–90% against severe disease
Poliovirus	Orimune; OPV; Poliomyelitis vaccine	Live attenuated trivalent, bivalent and monovalent polioviruses	Oral, 3 doses	Mucosal IgA and systemic IgG	Over 90% in most of the world
*Salmonella Typhi*	Vivotif; Ty21A	Live attenuated *S. typhi* bacteria	Oral, 3–4 doses	Mucosal IgA, systemic IgG and CTL responses	Variable, but more than 50%
*Vibrio cholera*	Dukoral; ORC-Vax; Shanchol	Inactivated *V. cholera* O1 classical and El Tor biotypes with or without CTB	Oral, 2–3 doses	Antibacterial, toxin-specific and LPS-specific IgA	Strong herd protection over 85%

Abbreviations: CTB, cholera toxin B subunit; CTL, cytotoxic T lymphocyte; LPS, lipopolysaccharide; OPV, oral polio vaccine.

**Table 2:** Characteristics of immune induction depending on the routes of mucosal immunization

*Route of immunization*	*Effective compartment*	*Form*	*Characteristics (advantage/disadvantage)*	*Examples*	*Reference*
Oral	Gastrointestinal tract, salivary gland, mammary gland	Liquid, pills	Enhances immune response both in systemic and mucosal areas; safe; easy to vaccinate; easy to scale up/induction of tolerance, the harsh environment of the gastrointestinal tract	Rotavirus, Poliovirus, *Salmonella typhi, Vibrio cholera,* Cholera toxin	^6^
Intranasal	Upper respiratory tract, genital tract	Sprays, drop	Enhances immune response both in systemic and mucosal areas; easy to vaccinate/side effects such as Bell's palsy, damage to nasal epithelium	Influenza type A, H1N1 influenza	^29^
Pulmonary	Respiratory tract	Aerosol, powders	Enhances immune response both in systemic and mucosal areas; easy to vaccinate, simplified logistics/requirement of device, difficulty in vaccination	Edmonston-Zagreb	^30^
Sublingual	Respiratory and gastrointestinal tracts	Liquid, pills	Quick diffusion into the venous circulation/lack of strong adjuvants, difficulty in vaccine formulation	HIV-1 gp41,	^31^
Intravaginal/Rectal	Genital tract	Cream	High relevance for sexually transmitted diseases/difficulty in inoculation	HIV-1, HSV-2	^32, 33^
Ocular	Ocular system	Drops	Generation of ocular mucosal immunity	HSV-2	^34, 35^

Abbreviation: HSV, Herpes simplex virus.

**Table 3:** M-cell-binding ligands and M-cell-specific molecules (modified from reference 60)

*Ligand*	*Receptors on M cells*	*Reference*
UEA-1	α1,2 Fucose	^56^
AAL	α-L-Fucose	^57^
Galectin-9	*N*-Glycans/repeated oligosaccharide	^58^
Peptide Co1 (SFHQLPARSPLP)	C5aR	^59^
Antibody NKM 16-2-4	α1,2 Fucose-containing carbohydrate	^54^
Antibody LM112	Sialyl Lewis A	^60^
Antibody 3G7-H9	Glycoprotein 2	^61^
σ1 protein (reovirus)	α2,3 Sialic acid	^62^
Invasion (*Yersinia*)	β1 Integrin	^63^
Long polar fimbriae (*E. coli, Salmonella*)	Unknown	^60^
FimH (*E. coli, Salmonella)*	Glycoprotein 2/uromodulin	^50^
OmpH (*Yersinia*)	C5aR	^59^
LPS	TLR-4	^64^
Lipoteichoic acid	TLR-2	^65^
Phosphorylcholine moiety of LPS	PAFR	^66^
Hsp60 of *Brucella abortus*	Cellular prion protein	^67^
Lipid A domain of LPS (Gram-negative bacteria)	AnxA5	^68^
Bacterial peptidoglycan	PGLYRP-1	^69^
SIgA	Unknown	^70^
c-Term domain of enterotoxin (*Clostridium perfringens*)	Claudin 4	^71^

Abbreviations: ALL, *Aleuria auranitia*; AnxA5, Annexin A5; PAFR, platelet-activating factor receptor; PGLYRP-1, peptidoglycan recognition protein-1; TLR, Toll-like receptor; UEA-1, *Ulex europaeus* 1.

**Table 4:** Mucosal adjuvants (modified from reference 75)

		*T-cell-mediated immune response*					
*Composition*	*Target*	*Th1*	*Th2*	*Th17*	*CTL*	*Mucosal IgA*	*Reference*
MDP	TLR-2	+	+			+	^65^
MPL	TLR-4	+			+	+	^78^
Flagellin	TLR-5	+			+	++	^79^
CT	GM1		+	+	+	++++++	^21^
CTA1-DD	Ig heavy chain	+	+	+	+	+++++	^80^
*Quillaja* saponins	DCs	+	+		+	++	^81^
Cationic DDA	DC uptake	+			+	++	^82^

Abbreviations: CT, Cholera toxin; DC, dendritic cell; DDA, dimethyldioctadecylammonium; MDP, muramyl dipeptide; MPL, monophosphoryl lipid A.
